# The welfare of wildlife: an interdisciplinary analysis of harm in the legal and illegal wildlife trades and possible ways forward

**DOI:** 10.1007/s10611-021-09984-9

**Published:** 2021-08-18

**Authors:** Tanya Wyatt, Jennifer Maher, Daniel Allen, Nancy Clarke, Deborah Rook

**Affiliations:** 1 Northumbria University, Newcastle upon Tyne, UK; 2 University of South Wales, Pontypridd, Mid Glamorgan UK; 3 Keele University, Newcastle-under-Lyme, UK; 4 London, UK

**Keywords:** Illegal wildlife trade, Legal wildlife trade, Green criminology, Animal welfare, Animal rights, CITES

## Abstract

Wildlife trade—both legal and illegal—is an activity that is currently the focus of global attention. Concerns over the loss of biodiversity, partly stemming from overexploitation, and the corona virus pandemic, likely originating from wildlife trade, are urgent matters. These concerns though centre on people. Only sometimes does the discussion focus on the wildlife traded and their welfare. In this article, we make the case as to why welfare is an important component of any discussion or policy about wildlife trade, not only for the interests of the wildlife, but also for the sake of humans. We detail the harm in the trade as well as the current welfare provisions, particularly in relation to the Convention on International Trade in Endangered Species of Wild Fauna and Flora (CITES), which guide global transport and trade. There are a number of ways that the current approach to wildlife welfare could be improved, and we propose ways forward in this regard.

## Introduction

Billions of wildlife^1^—from the entire range of species—are killed and captured every year as part of the global legal and illegal wildlife trades. Increasingly, in light of evidence on the links between zoonotic diseases (e.g., COVID-19) and biodiversity loss, the overexploitation of wildlife is identified as negatively impacting people living with and near the wildlife and their livelihoods. Compassion and respect for the wildlife harmed in these trades, however, is seldom a key focus. Taking a multidisciplinary approach our article explores whether and how space can be made for the welfare of wildlife in the ongoing debates on banning or regulating the legal trade and preventing and disrupting wildlife trafficking.

The wildlife trade—both legal and illegal—exemplifies how animals are exploited for human consumption and use with little discussion of animal welfare (and even less consideration for rights) [2]. At a time of heightened global awareness of, and concern for the plight of iconic species, there seems to be widespread ignorance or denial of the routine and serious harms experienced by all wildlife in these trades. This is despite an increased scientific basis evidencing animals are sentient and can therefore experience both pleasure and pain [3, 4], which has resulted in legislation protecting some animals from unnecessary suffering [5]. The evidence on how brutal wildlife trade and trafficking can be for the animals involved is extensive. Rhinos keen when they have been illegally shot so their horns can be cut off,their children have their spines cut with a machete or are taken captive [6]. Snakes are pumped full of water and starved for days before being skinned alive for the legal reptile leather industry [7]. Birds have their eyelids sown shut and are then stuffed into tubes to be smuggled across long distances, involving days without food and water [8]. Wildlife are left in snares and traps to die slowly from exposure, dehydration, and/or starvation, so people can have food, fur, or medicine [8]. It is commonplace for fatalities to occur during transportation, especially in the illegal trade [9]. Those animals who survive the illegal trade and are confiscated by law enforcement agencies, may be required for the duration of court cases or indefinitely to live in inadequate housing facilities [10], or upon being seized simply killed [11, 12]. For others who survive the trade and go on to live their life as a companion animal or in aquariums, farms, laboratories, and animal entertainment, it is extremely unlikely their behavioural and physiological needs will be met adequately in captive conditions. This can result in the abandonment and introduction of invasive species, who are often subsequently inhumanely destroyed as pests [9].

Most of the wildlife trade, which involves the sale or exchange of wild animals and plants, is legal, un- or partially regulated, and plays a fundamental role in our way of life and global economies. The illegal wildlife trade (also known as trafficking)—involving the capture, harvesting and trade of wild animals and plants contrary to national laws is estimated to be one of the largest global black markets [13]. Henceforth, when referring to both the legal and illegal trades we use the term (il)legal. Criminology scholars have highlighted the injury and suffering inherent in the (il)legal wildlife trades (see [2, 12, 14, 15] amongst others). In green criminological studies, it is well documented that many ‘legal’ practices can be as harmful, if not more harmful than those criminalised [16]. Animal geography and animal activist scholars suggest, from both an anthropocentric and a welfarist perspective, it seems that ‘unnecessary’ animal suffering is an unavoidable part of the (il)legal global wildlife trade, whether the wildlife is alive or killed [17, 18], leading some to demand a total ban on all global trade on animal welfare grounds [19]. It is evident from legal and critical animal studies that wildlife are viewed as human property and resources [1]. Despite the substantial evidence of cruelty in the (il)legal wildlife trade, little academic debate, or research addresses welfare in this rapidly growing area of concern. We propose this lack of regard for, or discussion of, welfare stems from humans viewing non-human animals as property rather than as individuals; objects not always as subjects; and as commodities not always as sentient beings.

In this article, we argue it is important to extend the current discussions on the wildlife trade to the individual abuse that wild animals suffer and to the structural violence [20] that they endure. It is crucial to include the legal trade in our discussion, due to the harms implicit in the commodification and exploitation of animals and the difficultly of differentiating between the (il)legal trades [21]. The social structures which systematically and routinely use wildlife and their bodies facilitate the structural violence under which these animals must live and die. Our paper adopts a transnational (e.g., focusing on English and international regulations) interdisciplinary approach to more fully and robustly evaluate whether it is important to include wildlife welfare in these debates and how we might go about doing this. By combining green criminology, animal geography, critical animal studies, applied animal welfare science, and legal studies, we intend to offer new ways forward. To do this, we have undertaken an interdisciplinary systematic literature review of the above-mentioned areas. These are synthesised into sections addressing why welfare is an important consideration, what welfare provisions are in place, and how to balance human and wildlife interests. We conclude with what possible next steps should be taken.

## Why welfare is an important consideration

The harms to animals in the (il)legal trade are evident to many, however, the paucity of research, legislation, and social condemnation in regard to the welfare of wildlife suggests it is important to start by making a case for the focus of this paper. There has been insufficient research conducted which focuses on the welfare implications for wildlife, even though as the trades grow, the associated harms increase. A review by Baker et al. [22], p. 931) of the available wildlife trade literature between 2006 and 2011 highlighted that articles were dominated by conservation issues, with conservation levers (arguments that might be used to influence a cause) being the most often mentioned (in 71% of 292 articles), and animal welfare considered in only a minority of articles. Species existence, biological fitness, and welfare in relation to health at the collective level are the concern of conservation. Animal welfare science provides the basis for which the welfare interests of individual animals and their species can be considered and protected [23]. Baker et al. [22] argue this emphasis on conservation not welfare is apparent in the monitoring and regulation of the wildlife trade. This is demonstrated by how international regulations—the Convention on International Trade in Endangered Species of Wild Fauna and Flora [CITES]—are interpreted and enforced (discussed further below). Furthermore, amongst the growing research on animal welfare and wildlife trafficking since 2011 (which we cite throughout), the continued overall absence of welfare in the literature is glaring. This may be because harm, that is, the subjective experience and physical welfare parameters of animals, can be difficult to observe and document in the real world as opposed to as part of scientific studies, and thereby may be underreported [22]. It is also possible that scholars avoid sensitive topics that can be unpopular and alienating to governments and other stakeholders (also discussed further below) as well as avoiding those topics that are low priority compared to other issues (i.e., human trafficking, terrorism and so forth). Nonetheless, the welfare issues are very real and affect both human and animals alike. This is clearly established in the World Health Organization [WHO] One Health [24] and the One Welfare [25] frameworks. Specifically, the harms wildlife are subjected to during and after (il)legal trade, and how human welfare and prosperity run in tandem with wildlife welfare are discussed below.

### Harm in the trade

The (il)legal wildlife trade can appear to be a victimless crime, but this anthropocentric view is only possible if not viewing animals as individuals, who experience and have an interest in avoiding suffering and pain [12]. Mellor et al. [26] categorise welfare into five domains: disease, injury or functional impairment,environmental challenges (such as temperature extremes and injurious structures or floors); behavioural or interactive restrictions; anxiety, fear, pain, or distress; and food and water deprivation or malnutrition. Importantly, their research focused on animals used in laboratory experiments. Arguably, the more extreme and covert conditions of capture, killing, or transportation, alongside the limited habituation to humans (in captive-bred animals) suggests additional welfare concerns for traded wildlife. Baker et al.’s [22] study found literature documenting harms to wildlife in each of Mellor et al.’s five domains. Of the 292 articles, 25% identified disease, injury, or functional impairment; 20% environmental challenges; 20% behavioural or interactive restrictions; 18% anxiety, fear, pain, or distress; and 13% food or water deprivation, or malnutrition. Behavioural or interactive restriction and anxiety, fear, pain, or distress were more often associated with wildlife being used alive. The impacts in the domains of disease, injury, or functional impairment, environmental challenge, and food or water deprivation or malnutrition were more likely to be reported when wildlife were captured, transported, and killed.

Baker et al.’s study found these harms may be underreported in general and particularly in international, illegal and wild-caught trade, and in the trade in reptiles. In terms of welfare, the study concludes that.

“greater attention should perhaps be paid to animals traded alive and in larger numbers (e.g., birds, reptiles, amphibians) and to those—including mammals—that are potentially subject to greater welfare impacts through live use, such as that driven by the demand for pets and entertainment” [22], p. 936).

Furthermore, low welfare standards during trade and trafficking can lead to higher mortality rates, which further compounds existing conservation problems of decreasing species’ population numbers. The mortality rate for some species, such as birds, is estimated to range from 30% to as high as 90% in the illegal trade [14, 27]. Despite wildlife dying in transit the costs can be absorbed by businesses and criminals [21]. Traders handling wildlife who are captured alive may allow them to suffer impacts associated with captivity, in the belief that sufficient numbers to make a profit will survive [21]. Inevitably, high rates of mortality in transit will trigger further wildlife capture to compensate for the loss in numbers, harming conservation efforts.

Welfare and conservation harms extend beyond the point of trade. The live animals removed and rescued from traffickers by enforcement agents can be re-victimised [10]. For example, Maher and Sollund [28], found that live animals confiscated and seized may be: (1) housed long-term in unsuitable accommodation; (2) rehomed to unregulated and unchecked organisations, which have returned them to the illegal trade; or (3) killed because there is nowhere suitable to home them. For those not euthanised, they will, with few exceptions, experience the rest of their lives in captivity. Few animals are repatriated to their country of origin. This is partially because 56% of live animal CITES seizures (between 2010 and 2014) occurred on import rather than export [29]. Repatriation of wildlife can be difficult (i.e., unknown country of origin), expensive (housing and transportation to their country of origin), and contrary to the welfare of the wildlife and species, who may not survive the journey or may introduce diseases to the wild. Wildlife destined for captivity, as pets, entertainment, zoos, or even in rescue centres, encompass all five welfare domain harms identified by Mellor et al. [26] and those commonly identified in domesticated animals. For example, an EU study found that 70% of animals die within 6 weeks at commercial supply houses and 75% of pet reptiles and amphibians die within the first year due to inappropriate welfare [9]. This report also highlighted the subsequent impact of invasive species, zoonotic diseases, and the economic consequences of the wild pet trade. Importantly, these post-trade issues are often state-sanctioned, structural and are frequently less understood and addressed than harms at other points in the trade. The welfare of wildlife, thereby, not only has serious implications for the animal individuals, but critically impacts species conservation, biodiversity, and human welfare and economies.

### Wildlife welfare is human welfare

The 2016 INTERPOL and UNEP [30] report links environmental crimes, such as wildlife trafficking, with other serious illegal activity, including corruption, counterfeiting, drug trafficking, cybercrime, and financial crimes as well as with terrorism and non-state armed groups. In addition to serious criminality and state security, the (il)legal trade impacts human security. Human security—a concept generally associated with the UNDP [31], p. 23) Human Development Report—“first, [requires] safety from such chronic threats as hunger, disease and repression. And second, it means protection from sudden and hurtful disruptions in the patterns of daily life—whether in homes, in jobs or in communities”. The welfare of wildlife—particularly the conditions in which they are caught, killed, transported, and kept—have direct and fatal consequences for human health and safety [24, 25]. The ongoing coronavirus pandemic is the result of the latest zoonotic disease—likely originating in bats, possibly passing through an unknown second species, and then on to humans through consumption [32]. COVID-19 follows a long list of zoonotic diseases (i.e., Severe Acute Respiratory Syndrome (SARS), HIV, Ebola—[33]). Zoonotic diseases are responsible for over two billion cases of human illness and over two million human deaths each year. Sixty percent (60%) of emerging infectious diseases are zoonotic and 70% of these are thought to originate from wild animals [34]. Wildlife trade and markets (such as the market in Wuhan, China possibly linked to the COVID-19 outbreak) are identified as unique epicentres for the transmission and mutation of potential viral pathogens [35–37]. Swift et al. [33] found that the diversity of species being brought into highly populated cities contributes to the likelihood of disease emergence. These wild animals are alive, so that they can be killed and delivered as fresh ‘products’ to consumers. Hundreds of individual wildlife of a variety of species are made more susceptible to diseases by being kept in close and stressful conditions, on an inappropriate diet, and exchanging excrement and viruses [32]. They are then butchered on the same surfaces, which exposes blood and organs to the viruses and are then consumed by people [32]. The welfare element is critical to the lack of hygiene in such places.

While the legal trade may regulate some aspects of animal welfare and hygiene, these are limited due to infrequent inspections, corruption, and the reliance on markets and companies’ self-regulation [9]. Furthermore, inspections will not identify all diseases as not all wildlife carrying disease will appear ill. The illegal trade is unregulated and lacks any of the above controls (e.g., not subject to inspections, quarantines, and other mechanisms for containing disease) [14]. Those not convinced welfare is important for the sake of the animal, may be persuaded of its importance because the physical health of wildlife can affect human health, especially those wildlife intended for consumption in our homes. Consequently, some non-governmental organisations [NGOs] are calling for a ban on wildlife markets [32, 37].

## Welfare provisions in place

Despite efforts to develop a universal declaration of animal welfare and rights,^2^ no international agreement exists on the standards of animal welfare for domesticated, captive, or wild animals. Broadly, there are three positions on how to approach animal welfare: property, welfare, and rights. After briefly detailing each of these, the paper examines the international framework of CITES which protects certain internationally-traded species from overexploitation to identify how each of these perspectives enter into the regulation and enforcement of the (il)legal trade. It is important to note that CITES is a trade, rather than a welfare convention, focused on international rather than domestic behaviours or markets and thereby, does not cover many aspects of the (il)legal use of wildlife. Nonetheless, we focus on it here as it is a significant multilateral agreement which provides the most coherent approach to the rules and standards of international wildlife trade.

### Property and welfare

While each country approaches animal protection differently, the most common view is of animals as property, commodities, and resources. Frequently, where animals are viewed as property, this is intertwined with a welfare-based approach. Consequently, we have merged our discussion here of these two approaches, using English^3^ legislation as an example.

Although under English law, wild animals (*ferae naturae*) are not deemed property, humans can acquire qualified property rights over them by capturing and taking them into their possession or if the wildlife moves onto privately owned land, they become the property of the landowner whilst present on the land [41]. According to UK legislation, humans enjoy absolute property rights over captive wildlife, however, domestic laws give protection from intentional and gratuitous pain and suffering in the form of criminal laws to prohibit ‘unnecessary suffering’. In English law, Sect. 9 of the Animal Welfare Act [42] (which applies to all “vertebrates other than man”) created a new ‘welfare offence’, which imposes a positive duty on the person responsible (possessing/controlling) for the wildlife to meet the animal’s welfare needs. These welfare needs are known as the five freedoms and comprise the following:

- need for a suitable environment,
- need for a suitable diet,
- need to be able to exhibit normal behaviour patterns
- any need to be housed with, or apart from, other animals, and
- need to be protected from pain, suffering, injury, and disease

Although domestic and captive animals should be protected from ‘unnecessary suffering’, the question of necessity is subjective and killing wildlife is still permissible, even if they are young and healthy, provided they are killed in a ‘humane’ way. This is enshrined in many other legal systems and, we suggest, is one of the reasons for suffering within the wildlife trade. Animals will never be respected as subjects, individuals, or sentient beings under this approach. As Rollin [, pp. 155–156] points out, animal protection and anti-cruelty laws “take the people who own or use animals as primary objects of moral concern, rather than the animals themselves”. From a geographical perspective, wildlife are generally considered as ‘lively commodities’, whose ‘value derives from their status as living beings’ ([, p. 2684], [17, 18, 43]). As living beings for sale, levels of welfare affect the monetary value of “products” [17, 44, 45].

### Rights and legal personhood

In contrast to both a welfare and a property-based approach, the rights perspective argues that humans should not have absolute rights over animals. This approach aims to protect wildlife from harm by criminalising their use and/or recognising their interests (e.g., not to experience pain). While no countries adopt a rights approach for all animals, there have been moves towards acknowledging human-like rights for some animals, in recognition that existing welfare statutes provide little protection [46]. In recent years, many countries and territories have passed laws or made constitutional changes to recognise the sentiency of animals. For example, Germany 1990, Switzerland 2000, the EU (Lisbon Treaty 2009), France 2015, Colombia 2015, and Spain 2018 [5] all have adopted legislation recognising animal sentience. There are obvious tensions between property-based and rights-based approaches to human-wildlife relations. At the core is the notion that human interest will always take precedent over wildlife interests [46]. That is particularly true if wildlife are characterised as unemotional objects. Acknowledging the sentience, personhood (which we return to later), and individuality of animals is central to the rights perspective [47]. There is considerable evidence using novel methodologies that shows a wide variety of wildlife across the spectrum of species are sentient, meaning not only that they can experience pain and suffering, but also feel positive emotions and pleasure [48]. As this becomes more widely known and accepted, it is becoming increasingly unacceptable to the public to exploit wildlife in ways that cause them to suffer unnecessarily. Nonetheless, no countries have bridged the sizeable gap between a rights approach and property and welfare perspectives.

#### CITES and welfare

In 2020, there were 183 parties to CITES, which regulates trade in 38,000 + species of animals and plants. These species can be legally traded if accompanied by the appropriate paperwork (e.g., permits). It is important to note that there are many more species taken from the wild, who are traded and not regulated by CITES. While some may be protected by domestic or other international conventions many are not. Further, as clarified above, CITES is a trade convention (e.g., to facilitate sustainable trade) not a welfare convention (e.g., to reduce harm to wildlife); the two are not synonymous. The CITES Preamble [49, no page] clearly identifies a human-centric property perspective to wildlife:

*“*Recognizing that wild fauna and flora in their many beautiful and varied forms are an irreplaceable part of the natural systems of the earth which must be *protected for this and the generations to come*;

Conscious of the *ever-growing value* of wild fauna and flora from *aesthetic, scientific, cultural, recreational and economic points of view*;

Recognizing that peoples and States are and should be the best protectors *of their own* wild fauna and flora;

Recognizing, in addition, that international co-operation is essential for the protection of *certain species* of wild fauna and flora against *over-exploitation* through international trade;” (*italics added*).

The word ‘welfare’ only appears once in the Convention text and only in reference to rescue centres having the authority to look after the welfare of species [49]. That is not to say welfare is completely absent. The language repeated throughout the Convention regarding exports is: “the Parties shall ensure further that all living specimens, during any period of transit, holding or shipment, are properly cared for so as to minimize the risk of injury, damage to health or cruel treatment” [49, no page]. Regarding imports, the wording is that the Management Authority of the importing country should ensure “the proposed recipient of a living specimen is suitably equipped to house and care for it” [49, no page]. These import welfare guidelines, however, only apply to Appendix I listed species, who are the most endangered species.

CITES provides guidelines for the welfare of wildlife in transit, which all parties must adopt and enforce to comply with the convention. In 2007, CITES adopted the International Air Transport Association [IATA] Live Animals Regulations [LAR] and in 2013 the Guidelines for the Non-Air Transport of Live Wild Animals and Plants (covering land and sea transport). Further, IATA [50] have introduced a voluntary program, which is designed to help companies improve their level of competency, infrastructure, quality management, and training relevant to the handling and transportation of live animals throughout their journey by air (e.g., cargo or cabin). These standards are set for the legal trade, the illegal trade has no such standards. IATA [51] has acknowledged the aviation industry has responsibilities in facilitating welfare in the (il)legal trade; it has not been made clear, however, how these responsibilities apply in practice. Article VIII (paragraph 4) of CITES explains how parties should “dispose” of confiscated wildlife (see also Resolution Conf. 10.7 (Rev. CoP15): Disposal of confiscated live specimens). Other than these provisions, the welfare of wildlife is a domestic concern—this includes how wildlife are captured, transported, cared for, and killed prior to export and their transit and care or “disposal” as specimens after import.

The terminology used within CITES—‘specimens’, ‘products’, ‘kilograms’, ‘units’ ‘parts’—objectifies wildlife. The annual and biennial reporting CITES requires from parties does not record deaths in transit or welfare violations. Further, there is no requirement to rehome, or repatriate confiscated animals though it is recommended as the first course of action [49]. According to Sollund and Maher [52] and Baker et al. [22], CITES parties pay little attention to welfare, as compared with the conservation requirements. Baker et al. [22] notes that of the recommendations made in the reviewed articles, 6% suggested making changes to CITES regarding welfare. They argue, CITES:

“is a potentially powerful and currently underused tool for improving animal welfare in the international wildlife trade…CITES has the capacity to reach many wildlife-trading countries and, potentially, to persuade members to adopt measures for improved animal welfare in wildlife trade” [22], p. 935).

Maher and Sollund [11] claim that because the trade is both legal and illegal it is difficult for CITES to convey a strong normative message to consumers and offenders through the application of laws and regulations. There is often a thin line between legal and illegal acts within the trade, which further blurs the message regarding the use and treatment of wildlife. Rather, the message conveyed through CITES, other regulations and the enforcement approach is that wildlife trafficking may be legally wrong, but the consumption and treatment of wildlife is acceptable. The sentience of wildlife is denied or ignored. This is because whenever wildlife becomes a commodity to be sold for profit, the legal protection exists for the commercial exploitation, not the welfare of the wildlife [1]. Consequently, welfare standards in the (il)legal trade are minimal and only in place to maximise profits and sustainability. Given the nature of the illegal trade, designed to evade customs and law enforcement, it is devoid of even the most basic regulatory standards and oversight. Rather, animals are placed in extremely abusive situations to facilitate the covert approach.

CITES [49] crucially recognises that communities interact, understand and value animals in a unique manner, based on a variety of socio-economic, cultural, and aesthetic factors. These parties also take a different approach to animal welfare—perceptions, policies and practices are not universal. The same is true for NGOs and local people. Some call for welfare improvements, others for the closure of live animal markets or an end to all trade [37]. Consequently, developing a more welfare-oriented conservation is fraught with challenges.

## Balancing human and wildlife interests

The differing perspectives are at the core of ongoing clashes and tensions within CITES. Addressing the (lack of) value placed on different species and adding further welfare considerations to CITES would fundamentally change the Convention, which raises important implications for key stakeholders. The (il)legal trade is motivated by a variety of factors. For some, it provides an essential livelihood, or a profitable business, while others are driven by the desire for luxury goods, beauty, and health [14]. Some stakeholders regulate and enforce a sustainable trade, while others campaign for and promote species and eco-justice, welfare, and rights. More broadly, we are all stakeholders in the trade, with some (indigenous) populations more critically affected by the consequences of unsustainable and illegal trade. Everyone who values wildlife and biodiversity—intrinsically or economically—is a stakeholder in the trade. Since the needs and motivations of stakeholders vary, efforts to improve welfare will inevitably alienate some stakeholders. At the heart of this problem is the perception that raising animal welfare will directly reduce the wellbeing of stakeholders reliant on the trade. However, using the proportionality approach, taken from human rights law, below we reconsider these areas of conflict. Proportionality is a balancing act whereby competing interests are weighed against each other [53–55].

### The effects of improving wildlife welfare on human stakeholders

When thinking about the conflicting interests of wildlife as resources versus wildlife as sentient beings, the concept of proportionality may be used to argue that some trade in wildlife is acceptable, but it must be proportionate. Therefore, welfare considerations must be weighed in the balance with trade considerations. As evidenced above, there are few welfare considerations in the regulation of the international trade. Wildlife welfare holds little, if any, weight in the balancing exercise. To identify how CITES might change, what follows is an example of how such a balancing exercise brought about changes in the German constitution. A ban on the religious slaughter of animals was challenged in the German Constitutional Court by a Muslim butcher. Despite the relevant welfare concerns, the court was required to reverse the ban due to the German Constitution, at the time, protecting a person’s professional freedom but not animal welfare. This case led to a change in the Constitution so that now animal welfare is a relevant consideration to weigh in the balance [56]. This does not mean that animal welfare is equal or superior to human rights, but it does mean that animal welfare must be weighed in the balance and cannot be ignored. A further consideration in weighing up the balance is the necessity and nature of the trade. The suffering experienced by the animals is seldom driven by need, rather, the (il)legal trade is driven by the desire for luxury goods, foods, and pets [22]. The nature of the wildlife trade has changed to improve hunting/capture efficiency and profits, resulting in the increased use of unsustainable techniques (e.g., hunting with semi-automatic weapons, long-line snaring). For example, the WWF [57] indicates that many fisheries throughout the world throw away more fish than they keep (i.e., bycatch). The suffering experienced by the billions of discarded fish and other animals in the trade, should be considered when weighing up the necessity of the trade and the needs of stakeholders in the trade.

Given the commercial value of the trade, many stakeholders benefit financially. Yet those achieving the most financial reward are the least likely to be dependent on wildlife trade for their livelihood; this is evident in that the profits at the end of the trade chain are the highest and being earned by businesses and organised crime networks [58]. However, the use of wildlife is a vital part of the livelihoods of millions of people, often in developing countries. Any changes to the trade must also consider these people and their rights and needs. Consequently, it is not enough to simply enhance wildlife welfare, it is also essential to focus on economic incentives that prioritise wildlife welfare, such as those that keep wildlife alive in the wild.^4^ Those who become stewards of nature deserve financial reward and effective protection. Furthermore, as mentioned, by creating alternative livelihoods for those people living alongside wildlife, harm to stakeholders can be minimised, while at the same time there may be considerable benefits (enhanced stability and biodiversity). If taking a proportionality approach, the harms are minimal especially when balanced against the benefits to wildlife and humans. Baker et al.’s [22] study found poaching removes key species from the environment, with negative implications for the ecosystem, conservation, wildlife welfare, and the livelihoods of current and future generations. A World Animal Protection investigation on jaguars exemplifies this [59, 60]. The practice is for hunters to shoot jaguars and capture and sell their cubs to businesspersons and criminals. The decline in jaguar populations can possibly result in an increase in rodents and herbivores, which in turn threatens to reduce the crop yields of farming communities and affecting livelihoods. The cost of the trade is unsustainably high—for both human and non-humans, both can benefit from an enhanced welfare response.

It need not be the case that bettering wildlife welfare must negatively impact human wellbeing; importantly, there are established links between the two [25]. The two movements ‘One Health’ and ‘One Welfare’ are complimentary projects that “highlight the interconnections between animal welfare, human wellbeing and the environment” [, no page]. On a global scale, according to UN (2020) and others’ calculations [25, 61], the COVID-19 pandemic could cost the global economy between USD 5.8 and 8.8 trillion and is triggering a global recession, business closures, mass supply chain disruption, and profit loss and forcing states to introduce costly stimulus packages. If this global economic loss is compared to the estimated global value of the legal trade in wildlife, of USD 300 billion [62], from a macro-economic perspective, the value of the global wildlife trade is not worth the risks it represents from a human health, animal welfare, and global economic perspective. As 56, p. 380) conclude “the associated costs of these preventive efforts would be substantially less than the economic and mortality costs of responding to these pathogens once they have emerged.”

### The alienation of stakeholders when addressing wildlife welfare

Considering the competing interests of animal and human stakeholders in the (il)legal trade, some stakeholders will be alienated by the call for enhanced animal welfare. Two particularly contentious areas are now discussed—the abuse of one animal in the protection others and a ban on all trade. Trophy hunting, and more recently canned hunting, have highlighted tensions in the wildlife trade in general and in CITES specifically. Proponents of trophy and canned hunting contend that the profits derived from killing a small number of iconic animals (e.g., rhinos, lions, giraffes) fund the conservation of other vulnerable species and support local people [63]. Opponents counter that trophy hunting normalises the killing of wildlife for entertainment and that the profits from the industry benefit a few (foreign) companies or line the corrupt pockets of rich locals and therefore, has limited benefits for conservation or local communities [64], Born [65]. As overexploitation and illegal trade in hunting trophies have increased, or at least become more widely know, there is growing pressure to stop this industry. The recent UK Government consultation on banning trophy hunting imports is an example of the direction of the conversation [66]. As previously stated, stakeholders are a diverse group and working with some stakeholders to influence and find common ground with other stakeholders is crucial to improving wildlife welfare. While it may not be possible to avoid alienating stakeholders supporting wildlife trade activities, this does not mean a welfare agenda should not be pursued.

One of the most contested approaches to wildlife protection is a ban on trade. This approach, only considered by CITES in the most extreme circumstances (e.g., species close to extinction), usually focuses narrowly on a specific species or location. Given the urgency required to protect some victims of the (il)legal trade, some argue that a total ban is needed [37] and would convey a stronger normative message to consumers and offenders, as has been done for rhino horn and ivory trades. While individual animals clearly benefit, it is unclear if bans would achieve sustainability for either wildlife or people in practice. This is due to the questionable planning and enforcement of such bans and the economic incentives in the trade, whereby bans may increase the value of endangered species and thereby increase profits and incentives for offenders [67]. For example, the decision by the Chinese Government to ban the sale of wildlife for food, in response to COVID, did not include a ban on the use of wildlife for medicine or pets. It is not clear what impact this ban has had on the (il)legal trades within and outside of China (e.g., the extensive markets for wildlife consumption as food in neighbouring Vietnam).

Rather than the ban itself, the way in which the message is delivered to stakeholders, may be the cause of alienation. For instance, Moorhouse et al. [68] found that the principle ethical arguments against ‘exotic’ pet ownership—species decline and poor welfare—are unlikely to significantly influence consumers. They propose that the personal cost of ignoring animal welfare may be a stronger message. The research found that “informing prospective exotic pet purchasers about either the zoonotic disease risks associated with, or potential illegality of, buying exotic pets could reduce consumer demand, potentially by up to 40%” [68], p. 342). Consequently, how welfare is justified—focusing on the mutual benefits of enhanced welfare in the trade to avoid costs that might directly affect them—is crucial. A similar argument may help avoid the alienation of business stakeholders, in that improving welfare reduces the risk to public health and inadvertently contributing to criminality. Whereby, some companies may identify enhanced welfare as a worthwhile cost to establish a reputation as principled, others may be more concerned with avoiding reputational loss because of engaging with unethical and illegal traders (e.g., money laundering, bribery, and corruption) [68]. Airlines can become signatories to the United for Wildlife Transport Taskforce [69] and help fight the illegal wildlife trade. Beyond this there are cases where a company takes extra measures such as declaring a global embargo on the transportation of wildlife hunting trophies, shark fin, and specific endangered species [70].

## Ways forward

There are numerous ways in which wildlife welfare might be improved in relation to the (il)legal trade. In this section, we focus on opportunities to improve welfare through current regulation and enforcement, focusing on CITES and enforcement practices (e.g., confiscation and transport). As part of this, we argue that improved welfare can be a means to improved enforcement.

### CITES

It is evident that CITES has failed to keep pace with the changes in animal (wildlife) law over the last 10 years. In recent years, there have been several cases in international courts challenging the legal status of wildlife and debating the controversial question of whether some captive wildlife can have rights and become legal persons. Personhood (being a legal person) is not the same as being a human although all humans are legal persons. A legal person is a legal construct and is defined as an entity that has capacity for legal rights [47]. For example, in 2017 New Zealand’s Parliament passed legislation granting legal personhood to the Whanganui river [71]. In the US, corporate personhood is recognised in law,however, the several cases since 2013 in which the Non-human Rights project has sought legal personhood for captive chimpanzees have all been unsuccessful [72]. In 2016, a judge in Argentina ruled that a captive chimpanzee, Cecilia, was a non-human legal person with “inherent rights” and granted a legal order called a ‘habeas corpus’ to transfer Cecilia from Mendoza Zoo, where she lived alone in a concrete enclosure, to a large chimpanzee sanctuary in Brazil (File No. P-72.254/15, Mendoza: 3 Nov 2016). The judge’s decision was based on her interpretation of Argentina’s General Environmental Law and, therefore, has not had wider impact on other habeas corpus cases overseas. More recently in 2020 the Islamabad High Court recognised that captive animals have rights and ordered the removal of animals from a zoo in Pakistan to sanctuaries (Islamabad Wildlife Management Board v Metropolitan Corporation Islamabad, W.P.No.1155/2019). This included two Himalayan brown bears and an elephant called Kaavan who had been gifted to the zoo in 1985 when he was 1 year old. Chief Justice Athar Minallah said that “animal species have rights and that it is a duty of humans to protect them” (at C.M.No.3976/2020, para.3).

While establishing legal personhood for animals remains problematic, evidence that animals are sentient and impacted by the (il)legal trade is not. Recognising the sentience of wildlife, as many countries now do, in CITES would be a significant step to protecting wildlife, the environment and people. Changes in law can help to change public opinion. For example, the decriminalisation of homosexuality or legalisation of same-sex marriages have led to a public that is more tolerant of the gay community (see [73] and [74] among others). Legitimacy through law encourages people to take an issue more seriously. If CITES were to stop referring to specimens and resources and instead talk about living, sentient beings, this could help to suppress consumer demand by encouraging greater respect for wildlife and their interests.

### Improving welfare in practice

While policy and legislative change requires time and can be difficult, there are more immediate practices in enforcement that could improve wildlife welfare. Foremost, the practice among government and enforcement stakeholders of holding confiscated wildlife in long-term captivity or euthanising them. While confinement and euthanasia are compatible with the policy of ‘taking care’ of wildlife, from species justice and legal proportionality perspectives, killing healthy animals is not a balanced approach, nor is it in the wildlife’s interest. Accountability is important in addressing the welfare balance (Maher and Sollund [28]). If CITES parties were required to report deaths during transportation, welfare provisions can be more adequately monitored and further provisions to reduce mortality put in place. CITES parties need to clarify their welfare responsibilities toward confiscated/seized live animals and provide transparency by way of an annual report on the outcome for all wildlife seized. If all parties made a formal commitment to protect confiscated/seized wildlife and to provide sufficient funding to ensure their welfare, euthanasia should only be required if there is a legitimate medical/welfare need.

As CITES iterates, repatriation, rather than destruction and captivity, should be the first course of action. Yet, the costs are prohibitive, as are the lack of facilities and expertise by CITES parties. In the interest of wildlife and conservation, there is a compelling case for exploring the use of financial instruments to support repatriation. Parties can charge more for CITES permits and set aside some of the proceeds to a repatriation fund. Illegal wildlife trade convictions should include the cost recovery for housing, care, and repatriation in the instances of live wildlife seizures, but in all cases asset forfeiture could be used to support repatriation. In addition to supporting repatriation, this, and all other mechanisms like confiscating proceeds of crime, could be used to fund prevention projects (such as keeping wildlife in the wild). A CITES resolution already exits (Rev. CoP15 Conf. 9.10) which states that when the Scientific Authority of the confiscating state deems it in the interest of the wildlife (specimen) to be returned, then such costs, and costs in relation to custody and destruction of the animal, shall be claimed from the trafficker. Resolutions are not mandatory, but further efforts to support members in implementing this resolution to support the welfare of wildlife in the trade and prevent future victims would be beneficial for all.

### Welfare as a tool for law enforcement

A welfare-based approach to the (il)legal wildlife trade is pragmatic if we want to improve not only the lived experiences of individual wildlife, but to combat the illegal trade, criminality, disease transmission, and insecurity. Attention to the welfare needs of trafficked wildlife can expose the modus operandi and become a tool for law enforcement. The clandestine nature of trafficking makes finding access points into the commodity chain challenging. According to D’Cruze et al. [75], the durability and survivability of species relates to mortality and morbidity, and this dictates the methods used by smugglers in the trade. Consequently, the ability to provide for wildlife welfare on the part of people transporting or smuggling wildlife dictates how and when they poach, smuggle, and sell that wildlife. D’Cruze et al. [75] provide two examples, the Indian Star tortoise and exotic birds to highlight how welfare influences the smuggling methods employed by criminals, and how each requires a different enforcement response.

African grey parrots, often caught to order for international trade, frequently die in transit. For subsistence-level hunters, ensuring survival is inconvenient and costly, and catching and exporting them requires significant preparation [76]. The modius operandi to avoid looking after the birds until shipment, requires organisation and planning, and there are fewer opportunities for law enforcement to catch offenders. In contrast, Indian Star tortoises are more easily and cheaply caught and kept, as they are hardier and can survive in terrible conditions. Consequently, tortoises may be opportunistically captured, may be held for extended periods by intermediaries until shipment and can be trafficked in a less sophisticated manner and without an organised infrastructure, whereas bird smuggling requires planning. Therefore, the criminal activity along the supply chain is partly dictated to by the welfare impacts on the wildlife in question, highlighting the importance of this often-overlooked component in responding to the illegal trade.

## Conclusion

In this article, we consider interdisciplinary perspectives and evidence on the importance of welfare in the wildlife trade and how this can be achieved. We argue that it is crucial *today* to extend the current discussion around the wildlife trade to the individual abuse and violence wildlife suffer and to the structural violence they endure. In doing so, we recognise this is likely to give rise to conflicts of interest between stakeholders (including animals), but this is not reason enough to ignore animal welfare. Rather, borrowing the concept of proportionality from human rights law, we suggest weighing competing interests to find a more appropriate balance. Using this approach, one position could be that some trade in wildlife is acceptable, but if so, it would need to be proportionate. Welfare considerations must be weighed in the balance with trade considerations and consumer needs. As the trade currently stands, we propose that there are entry points within it where standards of welfare can be introduced, the lived experiences of individual animals can be improved, and all stakeholders can benefit. To achieve this, it is important to address how animals are viewed and referred to in the trade—as objects seldom subjects; as property seldom individuals; and as commodities seldom sentient beings. A welfare approach should not be seen or used in isolation, it should be viewed as a collaborative tool, which can strengthen all responses to, including a ban on, the (il)legal trade.

Despite global calls at the highest levels of society to protect wildlife, their welfare needs are seldom high on the agenda. Failure to address these needs is a concern for the sustainability of the trade, conservation and human health and security. Stakeholders, therefore, must find ways of minimising such suffering. Moving welfare onto the political wildlife trade agenda requires more and better interdisciplinary scientific evidence, and wildlife welfare needs to be seen not as an isolated peripheral interest, but as associated with wider concerns that conspicuously affect our collective future [22]. This could not be timelier considering the COVID-19 pandemic. There is a paucity of research both in the natural/life sciences and social sciences on the welfare impacts on non-human and human animals of the (il)legal wildlife trade. Further research (including animal welfare scientists, veterinarians, criminologists, lawyers, behavioural science researchers, economists, etc.) is required to ensure future policy is scientifically informed and stakeholders can be educated. More evidence is needed to craft the right messages, supported by key stakeholders (e.g., veterinary, and human health professions) that demonstrates the welfare impacts of capture and captivity on animals and their environment, raises concerns over the safety of keeping and consuming animals, and highlights the criminality in the trade, may reduce demand. Regulation and enforcement are vulnerable to the limitations of data availability. This can be improved by demanding welfare data be collected and publicised, promoting collaboration and transparency among CITES parties.

Embedding a welfare approach will bridge property-based and rights-based perspectives and bring the regulation of the trade in line with current views of animals. In the current climate in animal law and ethics, CITES is outdated by giving so little weight to welfare. And though altering CITES might seem unlikely or too difficult, there is recognition by CITES experts that most of the parties largely support more efforts to improve animal welfare and that this is the direction that the Convention needs to move in [77]. Even those not convinced by a welfare argument should be swayed by the futility of allowing billions of needless deaths in the trade and the death or long-term captivity of wildlife upon removal from the illegal trade, especially considering recent pledges to respond to the illegal trade and loss of biodiversity. CITES, and national governments, should recognise animal sentience, in line with the EU and many other countries, and start preparing for a time when some wildlife, such as great apes, are deemed legal persons with a right to bodily liberty. In the absence of CITES recognising animal sentience and developing its welfare credentials, stakeholders can move to support the development of a Universal declaration of animal sentience/welfare (see Footnote 2).

In line with green criminology’s species justice perspective [78], it is important to acknowledge animals as victims of welfare violations in the legal trade and as victims of trafficking. We should also recognise the capacity of wildlife to suffer harms such as deprivation of freedom, natural behaviours, and associations [1], and that by improving welfare, we are improving human lives. In doing so, species justice is possible, if combined with effective regulation and enforcement which provides individual and systemic protection [78] for all animals.

## Data Availability

All materials cited herein are publicly available.

## Footnotes

1 For simplicity we use the term wildlife or animal throughout the paper whilst acknowledging that the vocabulary is contentious (see [1])

2 see UNESCO Universal Declaration of Animal Rights [38] (http://www.esdaw.eu/unesco.html), Universal Declaration of Animal Welfare [39] (https://www.worldanimalprotection.ca/our-work/global-animal-protection/universal-declaration), Declaration of Animal Rights [40] (http://declarationofar.org/), American Bar Association proposed international convention on animal welfare (https://www.americanbar.org/content/dam/aba/administrative/news/2021/02/midyear-resolutions/101c.pdf) and Global Animal Law Association proposed UN Convention on Animal Health and Protection (https://www.uncahp.org/).

3 While animal welfare legislation is devolved in the UK and varies in England, Wales, Scotland and Northern Ireland, the majority of the points here refer to all four countries that make up the UK.

4 If animal welfare is the focus, the most effective and vital point of intervention is where animals are removed from the wild, as this is when animals are most likely to be returned to their natural environment and are least likely to have experienced the various harms in the trade. However, few resources are available in most source countries to successfully repatriate/return victims of the trade [11].
